# Supplementary material for: A Randomized Controlled Study to Evaluate the Safety and Reactogenicity of a Novel rVLP-Based Plant Virus Nanoparticle Adjuvant Combined with Seasonal Trivalent Influenza Vaccine Following Single Immunization in Healthy Adults 18–50 Years of Age
Source: Vaccines (Basel). 2020 Jul 20;8(3):393. doi: 10.3390/vaccines8030393 (PMC7564144; doi:10.3390/vaccines8030393)
Supplement: Supplementary file 1 [file vaccines-08-00393-s001.pdf]

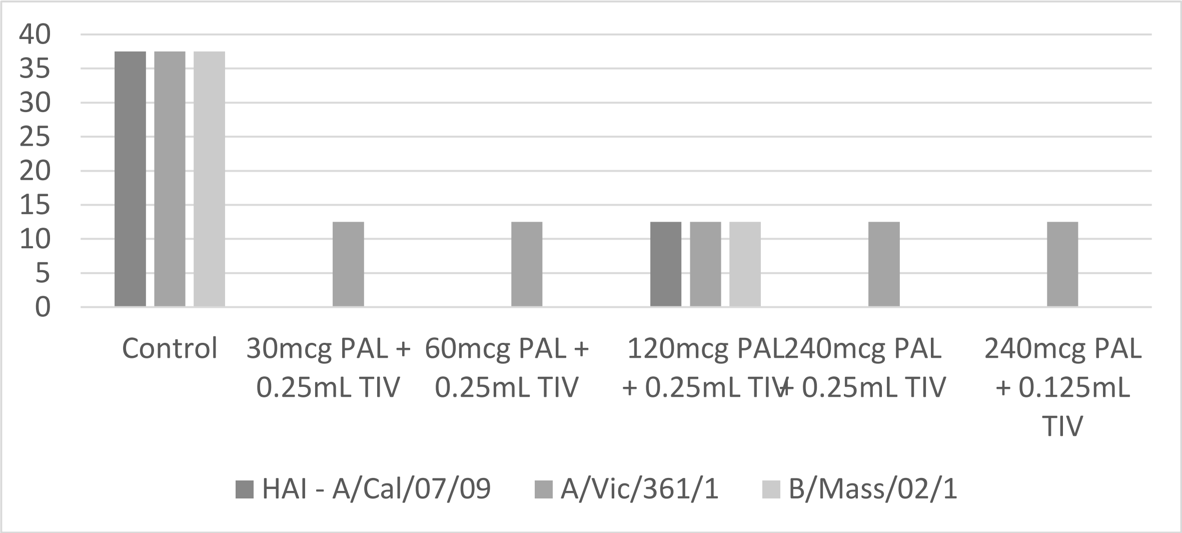

Supplemental Figure 1. Percentage of participants with 4-fold antibody rise of HI titer at Day 28

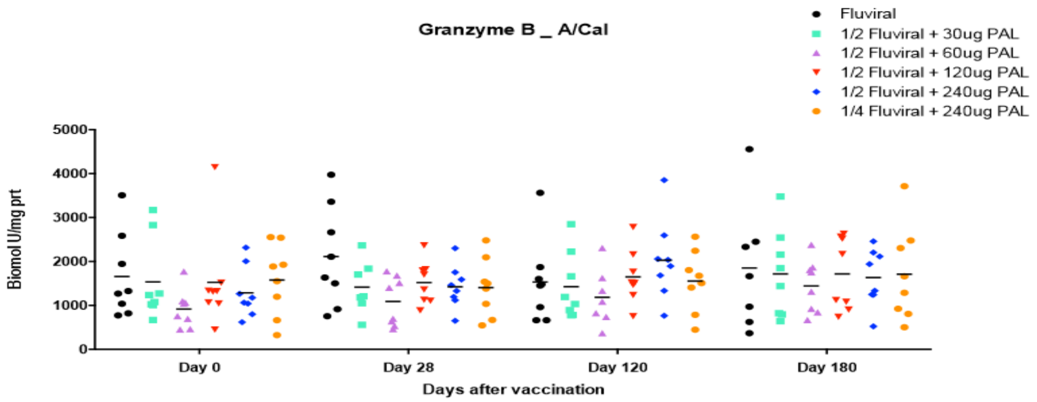
Granzyme B _ A/Cal
Fluviral
1/2 Fluviral + 30ug PAL
1/2 Fluviral + 60ug PAL
1/2 Fluviral + 120ug PAL
1/2 Fluviral + 240ug PAL
1/4 Fluviral + 240ug PAL
Biomol U/mg prt
5000
4000
3000
2000
1000
0
Day 0
Day 28
Day 120
Day 180
Days after vaccination

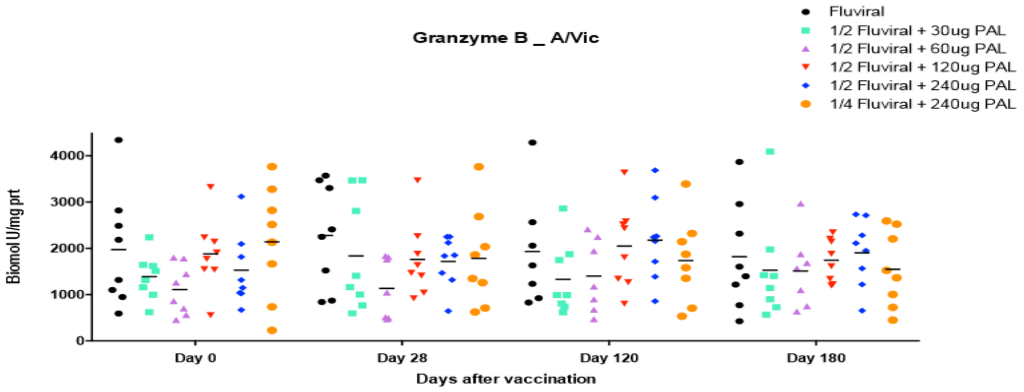
Granzyme B _ A/Vic
Fluviral
1/2 Fluviral + 30ug PAL
1/2 Fluviral + 60ug PAL
1/2 Fluviral + 120ug PAL
1/2 Fluviral + 240ug PAL
1/4 Fluviral + 240ug PAL
Biomol U/mg prt
4000
3000
2000
1000
0
Day 0
Day 28
Day 120
Day 180
Days after vaccination

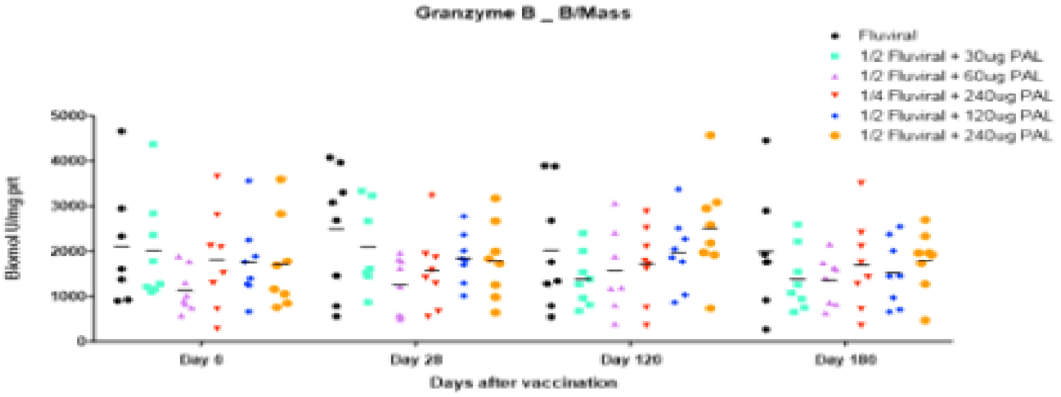
Granzyme B _ B/Mass
Fluviral
1/2 Fluviral + 30ug PAL
1/2 Fluviral + 60ug PAL
1/4 Fluviral + 240ug PAL
1/2 Fluviral + 120ug PAL
1/2 Fluviral + 240ug PAL
Biomol U/mg prt
5000
4000
3000
2000
1000
0
Day 0
Day 28
Day 120
Day 180
Days after vaccination

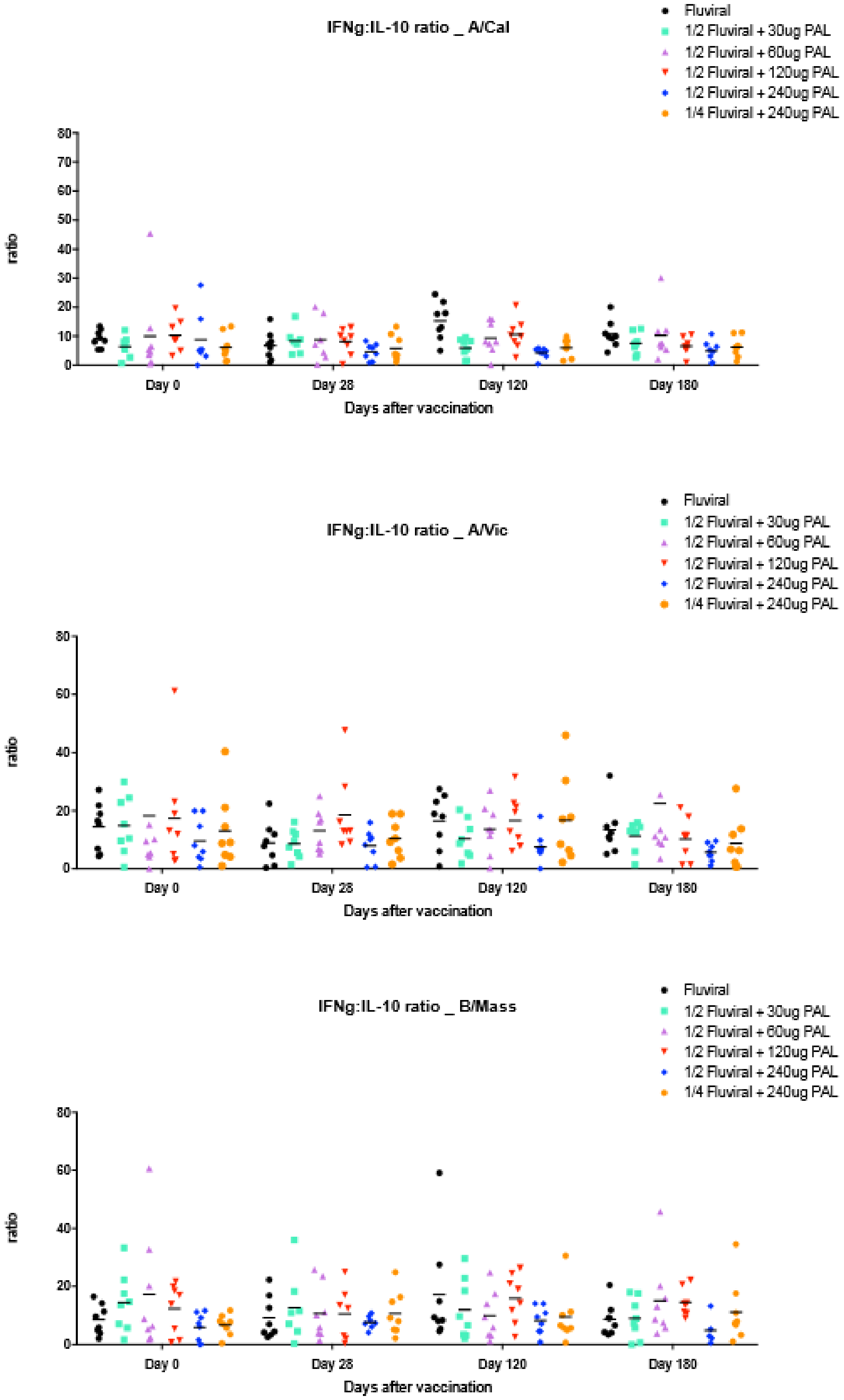

Supplemental Figure 2. Cell mediated Immune responses (Granzyme B and IFN- :IL-10 responses to three influenza strains in TIV).

Supplemental Table 1. Adverse event Intensity Grading

Adverse Event	Intensity grade	Parameter
Pain at injection site	0	None
	1	Mild: Any pain neither interfering with nor preventing normal every day activities.
	2	Moderate: Painful when limb is moved and interferes with every day activities.
	3	Severe: Significant pain at rest. Prevents normal every day activities.
Redness at injection site or		Record greatest surface diameter in mm
Swelling at injection site		Record greatest surface diameter in mm
	0	≤ 20 mm
	1	> 20 mm to ≤ 50 mm
	2	> 50 mm to ≤ 100 mm
	3	> 100 mm
Fever*		Record temperature in °C/°F
	0	< 37.5 °C/ 99.5°F
	1	≥ 37.5 °C/ 99.5°F to ≤ 38.5 °C/ 101.3°F
	2	> 38.5 °C/ 101.3°F to ≤ 39.5°C/ 103.1°F
	3	> 39.5°C/ 103.1°F
Headache	0	Normal
	1	Mild: Headache that is easily tolerated
	2	Moderate: Headache that interferes with normal activity
	3	Severe: Headache that prevents normal activity
Fatigue	0	Normal
	1	Mild: Fatigue that is easily tolerated
	2	Moderate: Fatigue that interferes with normal activity
	3	Severe: Fatigue that prevents normal activity
Gastrointestinal symptoms	0	Gastrointestinal symptoms normal
(nausea, vomiting, diarrhea and/or abdominal pain)	1	Mild: Gastrointestinal symptoms that are easily tolerated
	2	Moderate: Gastrointestinal symptoms that interfere with normal activity
	3	Severe: Gastrointestinal symptoms that prevent normal activity
